# Overall survival of patients with chemotherapy-naive advanced melanoma treated with ipilimumab 3 mg/kg in clinical trials

**DOI:** 10.1186/1479-5876-12-S1-P8

**Published:** 2014-05-06

**Authors:** Reinhard Dummer, Dirk Schadendorf, Paolo A Ascierto, James Larkin, Celeste Lebbé, Axel Hauschild

**Affiliations:** 1 University Hospital Zurich, Zurich, Switzerland; 2 University Hospital Essen, Essen, Germany; 3 Istituto Nazionale Tumori Napoli, Fondazione G. Pascale, Napoli, Italy; 4 Department of Medicine, The Royal Marsden Hospital, London, UK; 5 Department of Dermatology, APHP St Louis Hospital, University Paris Diderot, Paris, France; 6 University Hospital Schleswig-Holstein, Campus Kiel, Kiel, Germany

## Background

Dacarbazine (DTIC) is currently the only agent approved throughout Europe for patients with previously untreated, advanced melanoma that is not restricted by tumour genotype (e.g. BRAF status). Based on recent trials in patients with previously untreated, advanced melanoma, median overall survival (OS) and a 1-year OS rate of approximately 9 months and 36% represent conservative, historical benchmarks for DTIC monotherapy [1–5]. Data from clinical trials and expanded access programmes show that ipilimumab treatment consistently provides a proportion of patients with long-term clinical benefit, irrespective of whether they are pretreated or treatment-naive. In Europe, ipilimumab is indicated for use in adult patients who have received prior therapy. To support the use of ipilimumab in the previously untreated setting, data were analysed from ipilimumab clinical trials.

## Materials and methods

Data were pooled from chemotherapy-naive patients who received ipilimumab 3 mg/kg as monotherapy in one of four randomised clinical trials: MDX010-20 (NCT00094653), a phase 3 trial of ipilimumab alone or in combination with gp100 vaccine versus the vaccine alone; MDX010-08 (NCT00050102), a phase 2 multi-dose study of ipilimumab with or without dacarbazine; CA184-004 (NCT00261365), a prospective phase 2 biomarker study, and CA184-022 (NCT00289640), a phase 2, dose-ranging study. Details on these four trials have previously been published [6–9]. Patients received ipilimumab 3 mg/kg x 4 doses with the option for retreatment (MDX010-20) or maintenance therapy (CA184-004/022).

## Results

Of the patients who received ipilimumab 3 mg/kg within one of the four clinical trials described above, 78 had not received prior chemotherapy and were eligible for analysis. Of these, 27% had elevated lactate dehydrogenase levels at baseline and 53% had received prior immunotherapy for advanced melanoma. With a median follow-up of 11.6 months (range: 0.03–69.7), median overall survival was 13.47 months (95% confidence intervals [CI]: 11.2–19.6) and the one- and two-year survival rates were 54.1% (95% CI: 42.5–65.6) and 31.6% (95% CI: 20.7–42.9), respectively. Survival outcomes compared favourably to historical observations with dacarbazine in phase 3 trials of patients with previously untreated, advanced melanoma.

## Conclusions

Therapeutic options for treatment-naïve patients remain limited. OS data from chemotherapy-naive patients **368/400 words)**
